# Organic-phase synthesis of Li_3_V_2_(PO_4_)_3_@Carbon nanocrystals and their lithium storage properties

**DOI:** 10.1039/c8ra02490a

**Published:** 2018-05-25

**Authors:** Cunliang Zhang, Yanmei Liu, Jian Li, Kai Zhu, Zhe Chen, Shijun Liao, Xinhe Zhang

**Affiliations:** Mcnair Technology Co., Ltd Dongguan 523800 China zcliang@126.com; School of Chemistry and Chemical Engineering, South China University of Technology Guangzhou 510641 China; Department of Automobile Engineering, Shangqiu Polytechnic Shangqiu 476000 China; Shangqiu Medical College Shangqiu 476000 China

## Abstract

Decreasing particle size is an efficient strategy for improving the lithium storage properties of Li_3_V_2_(PO_4_)_3_ (LVP) due to a shorter transport distances of lithium ion and electrons. However, designing and synthesizing LVP nanocrystals (NCs) with sizes smaller than 30 nm remains a challenge. In this work, we developed a facile approach for the fabrication of the monodisperse LVP NCs through a robust high-temperature organic-phase method. The thermodynamics of the synthesis and the possible reaction mechanism were investigated. The results indicate that the organic-phase environment (at 320 °C) may not thermodynamically allow the crystallization of LVP. Nevertheless, oleic acid (OA) and oleylamine (OAm) are essential as capping agents to hinder the agglomeration and growth of the particles. Based on the thermodynamic need, calcination is essential to prepare LVP. The surface electronic conductivity of the LVP NCs was enhanced through a subsequent carbon-coating treatment. The optimum combination of reduction and carbon coating is very favorable for the kinetics of electron transfer and lithium ion diffusion. Therefore, the fabricated LVP@C NCs exhibit superior lithium storage properties with excellent rate capability (84 mA h g^−1^ at a rate of 20C) and perfect cyclic stability (96.2% capacity retention after 200 cycles at 5C), demonstrating their potential application in high-performance lithium-ion batteries.

## Abstract 2

Li_3_V_2_(PO_4_)_3_@Carbon nanocrystals exhibit superior lithium storage properties due to the shortened lithium-ion diffusion length and the enhanced surface electronic conductivity.

## Graphical abstract

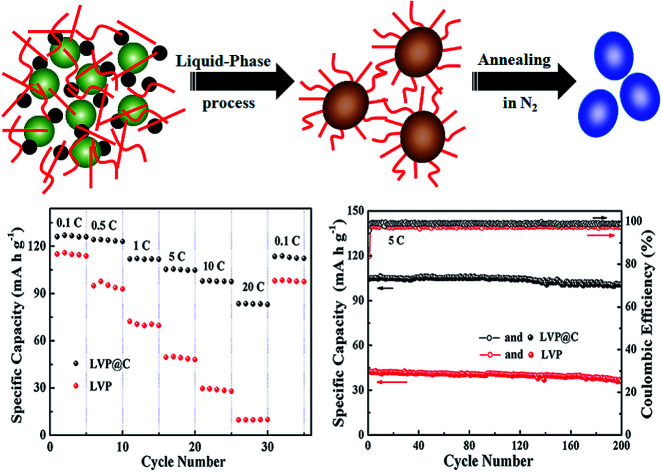

## Introduction

Rechargeable lithium-ion batteries (LIBs) have been rapidly developed for applications in portable electronics, hybrid electric vehicles (HEVs), electric vehicles (EVs), and smart grids owing to their high energy density.^1–3^ Cathode materials play a key role in high-performance LIBs.^4^ Recently, monoclinic lithium vanadium phosphate, Li_3_V_2_(PO_4_)_3_ (LVP), a phosphate compound with a sodium super-ionic conductor (NASICON) framework, has been considered as a promising cathode material due to its high operating voltage, large theoretical specific capacity, structural stability and abundant reserves.^5–10^

However, the poor electronic conductivity (about 2.3 × 10^−8^ S cm^−1^) and the slow lithium ion diffusion of LVP limit its practical applications.^11,12^ Carbon coating is an effective way to remarkably enhance the surface electronic conductivity and improve the specific capacity of LVP.^5,13–16^ To further improve rate capability, the ion diffusion kinetics need to be enhanced. In accordance with the diffusion formula *t* = *L*^2^/*D* (where *t* is the ion diffusion time, *L* is the ion diffusion distance and *D* is the ion diffusion coefficient), further reducing the particle size is an effective strategy to shorten the ion diffusion length, thereby accelerating lithium ion transfer in LVP and greatly improving the rate performance.^15,17–20^ Shen *et al.* reported that Li_4_Ti_5_O_12_/carbon core–shell nanostructured electrode showed superior lithium intercalation properties because of the sizes of the refined active particles and the good conductive medium of carbon.^21^ Fei *et al.* also reported that LVP/carbon electrode materials with hierarchically porous structures had exceptional cycle stability and excellent high-power capability; however, the synthesis of these materials is intricate.^14^ The prepared LVP particles generally aggregate easily *via* solid-phase reaction and the sol–gel process, which results in enlarged crystallite size and increased lithium ion diffusion length. Hydrothermal and solvothermal methods are carried out in a closed container and have high temperature and high pressure equipment requirements. Therefore, it is challenging but significant to develop a facile and low-cost strategy for synthesizing nanosized LVP electrodes with perfect lithium storage performance.

Oleic acid (OA) and oleylamine (OAm) are high-boiling-point (350 °C) organic solvents that can be used to synthesize nanosized materials.^22–27^ On one hand, they provide a high-temperature environment to facilitate nucleation. On the other hand, OA and OAm work as capping agents to limit the sizes of crystal nuclei to the nanometer scale. Hou *et al.* reported that uniform FeO nanoparticles were prepared through the decomposition of iron(iii) acetylacetonate in a high-temperature environment provided by OA and OAm as a solvent system.^27^ Doi *et al.* reported that monodisperse nanosized LiMnPO_4_ particles synthesized using a similar preparation method had poor electrochemical properties for lithium-ion batteries.^24^ Jiang *et al.* enhanced the electronic conductivity of nanosized LiFePO_4_ through a carbon-coating treatment and improved its lithium storage properties.^23^

In this work, a robust and simple liquid-phase method was applied to synthesize LVP nanocrystals (NCs) for high-power lithium-ion batteries through a low-cost and environmentally friendly process. Both OA and OAm served as solvents and surfactants and provided a high-temperature environment.^23,26,27^ The sizes of the obtained LVP NCs are around 30 nm. Through the *in situ* polymerization of dopamine and carbonization, LVP@C NCs were then synthesized. The structures and lithium storage properties of the as-prepared LVP and LVP@C NCs were investigated.

## Experimental

### Synthesis of LVP NCs

All reactants and solvents were of analytical grade and used without further purification. In a typical procedure, vanadium(iii) acetylacetonate (1 mmol), lithium acetate (1.5 mmol) and phosphoric acid (1.5 mmol) were first added into a mixed solvent of OA (30 mL) and OAm (30 mL) in a three-necked flask followed by stirring and dissolution. The solution was then dehydrated at 120 °C for 1 h under nitrogen atmosphere. Subsequently, the system was heated to 320 °C at 10 °C min^−1^ and kept at this temperature for 1 h under nitrogen flow. After cooling naturally, the brown precipitate (hereafter denoted as the intermediate phase) was harvested by centrifugation and washed thoroughly with ethanol and hexane several times. Finally, the intermediate phase was sintered at 700 °C for 2 h in nitrogen atmosphere to produce the LVP NCs.

### Synthesis of LVP@C NCs

Typically, 100 mg of the as-prepared LVP NCs were dispersed in tris-buffer (50 mL, pH = 8.5) solution, and a suspension was formed through ultrasonic treatment for 30 min. Subsequently, certain amounts of dopamine were added to the suspension under constant stirring for 2 h at room temperature. The precipitate was then collected by centrifugation and dried at 60 °C in a vacuum oven. Finally, the powder was annealed under nitrogen atmosphere at 500 °C for 2 h to obtain LVP@C NCs.

### Material characterization

The X-ray diffraction (XRD) patterns of the intermediate phase and LVP NCs were obtained using a Bruker D8 Advance X-ray diffractometer with Cu Kα radiation. Scanning electron microscopy (SEM) was performed using a JEOL JSM-7000 field-emission scanning electron microscope. Transmission electron microscopy (TEM) was conducted on JEOL JEM-2100 and PHILIPS TECNAI-12 transmission electron microscopes. The differential scanning calorimetry (DSC) curves of vanadium(iii) acetylacetonate were collected on a NETZSCH STA 409 PC calorimeter. The Fourier transform-infrared (FT-IR) spectra of the intermediate phase and LVP were measured using a NEXUS670 spectrometer.

### Electrochemical measurements

Electrochemical tests were carried out by galvanostatic cycling in CR2016-type coin cells. Briefly, 80 wt% active materials, 10 wt% carbon black and 10 wt% polyvinylidene fluoride (PVDF) binder were mixed in *N*-methyl pyrrolidinone (NMP) to obtain a slurry. The slurry was then uniformly deposited onto an aluminum foil current collector. Finally, the electrodes were dried at 110 °C for 12 h in a vacuum oven. Test cells were assembled in an argon-filled glove box using metallic lithium foil as the counter and reference electrodes and a polypropylene membrane (Celgard 2400) as a separator. A 1 M solution of LiPF_6_ dissolved in ethylene carbonate (EC)/dimethyl carbonate (DMC) with a volume ratio of 1 : 1 was used as the electrolyte. Galvanostatic charge/discharge experiments were performed using a CT2001A cell test instrument (LAND Electronic Co.) at different current densities between 3.0 and 4.3 V (*vs.* Li/Li^+^). All the measurements were carried out at ambient temperature.

## Results and discussion

**Fig. 1:**
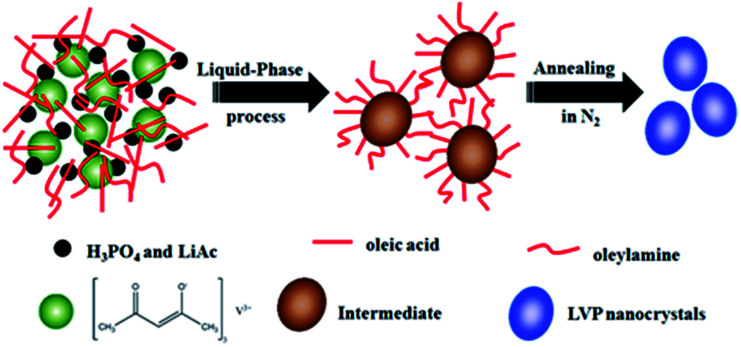
Principle schematic illustration of the fabrication of LVP NCs.

**Fig. 2:**
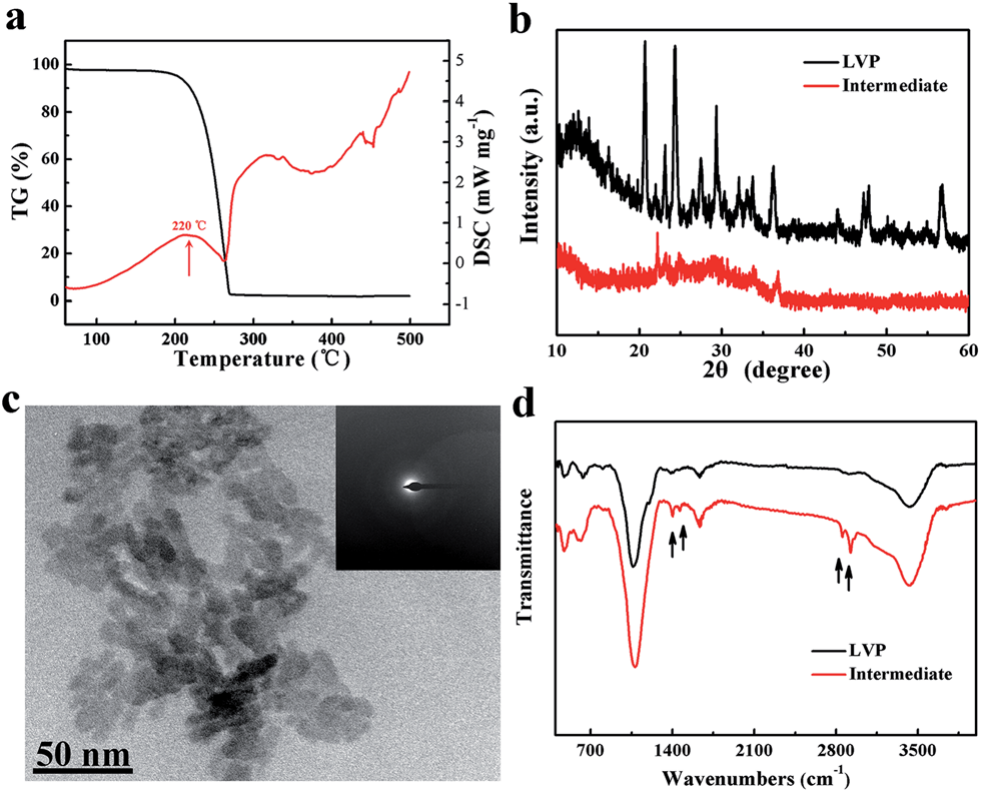
(a) DSC curves of vanadium(iii) acetylacetonate at 10 °C min^−1^ under nitrogen. (b) XRD patterns of the intermediate and LVP. (c) TEM image of the intermediate and the corresponding SAED pattern (inset). (d) FT-IR spectra of the intermediate and LVP.

A schematic of the synthesis of LVP NCs is illustrated in Fig. 1. Vanadium acetylacetonate as a vanadium precursor decomposes at about 220 °C under nitrogen atmosphere (as shown in the DSC curves in Fig. 2a). In this experiment, the vanadium precursor was at 320 °C because of the mixed organic solvent consisting of OA and OAm.^28^ Therefore, the vanadium precursor transformed into vanadium oxide as a core. Furthermore, the OA and OAm molecules covered the surfaces of the LVP precursors as capping agents and confined the growth of the core during the reaction process. The intermediate phase was extracted. Finally, LVP NCs were obtained *via* annealing under nitrogen.

The XRD patterns of the intermediate phase and as-synthesized LVP NCs are presented in Fig. 2b. From the tiny diffraction peaks of the intermediate phase, we conclude that it is a mixture of Li_3_PO_4_ (JCPDS no. 15-0760) and an amorphous component.^29^ According to previous reports,^30^ the hydrothermally treated precursors were converted into amorphous vanadium phosphorus oxide (VPO_4_) at 300 °C. We conjecture that the amorphous component may be amorphous VPO_4_ in this experiment, similar to in previous reports.^29,31^ The morphology and nanostructure of the intermediate phase were further investigated by TEM and selected-area electron diffraction (SAED), as shown in Fig. 2c. The morphology was indistinct, and the SAED pattern of the intermediate showed broad, diffuse rings. These results further confirmed the amorphous nature of the intermediate, which is apparently different from the LiFePO_4_ and LiMnPO_4_ prepared using a similar preparation method.^23,24^ The energy may not be sufficient to crystallize LVP. Thus, we obtained well-crystallized and phase-pure LVP with a monoclinic structure (space group: *P*2_1_/*n*; JCPDS no. 01-072-7074; lattice parameters: *a* = 7.741 Å, *b* = 7.684 Å, *c* = 6.754 Å, β = 84.929°) after calcination at 700 °C for 2 h (Fig. 2b). Using Scherrer's formula based on the (121) peak, the grain size of the LVP NCs was estimated to be 29 nm. These results show that the organic-phase environment (at 320 °C) may not thermodynamically allow the crystallization of LVP. Nevertheless, this environment is essential to provide a capping agent to hinder the agglomeration and growth of the particles.

According to the above XRD and TEM analysis, we deduce that the possible chemical reaction during organic-phase synthesis at 320 °C follows the route:

1 V(C_5_H_7_O_2_)_3_ + 3C_2_H_3_O_2_Li + 2H_3_PO_4_ → VPO_4_ + Li_3_PO_4_ + Ca–H–O

Hereafter, C–H–O was the residue during the reaction process.

To meet the thermodynamic requirements, calcination is essential to prepare LVP. During the calcination process, the chemical reaction taking place presumably follows the route:

2 2VPO_4_ + Li_3_PO_4_ → Li_3_V_2_(PO_4_)_3_

As shown in Fig. 2d, the FT-IR spectra of the intermediate component and the as-synthesized LVP NCs were measured. The FT-IR spectrum of the intermediate component is very similar to that of the LVP NCs with the exceptions of the peaks at around 1400, 1630, 2850 and 2920 cm^−1^. The bands near 1400 and 1630 cm^−1^ belong to the symmetrical stretching vibration and asymmetrical stretching vibration of the carboxy group.^24,32^ Moreover, the bands at about 2850 and 2920 cm^−1^ are attributed to the C–H stretching vibrations of the methylene and methyl groups.^33^ These results demonstrate that residual organic compounds from the OA and OAm solvent system exist on the surfaces of the intermediate components. Therefore, we conclude that OA and OAm attached the precursor and suppressed the agglomeration of nanoparticles during the reaction.

**Fig. 3:**
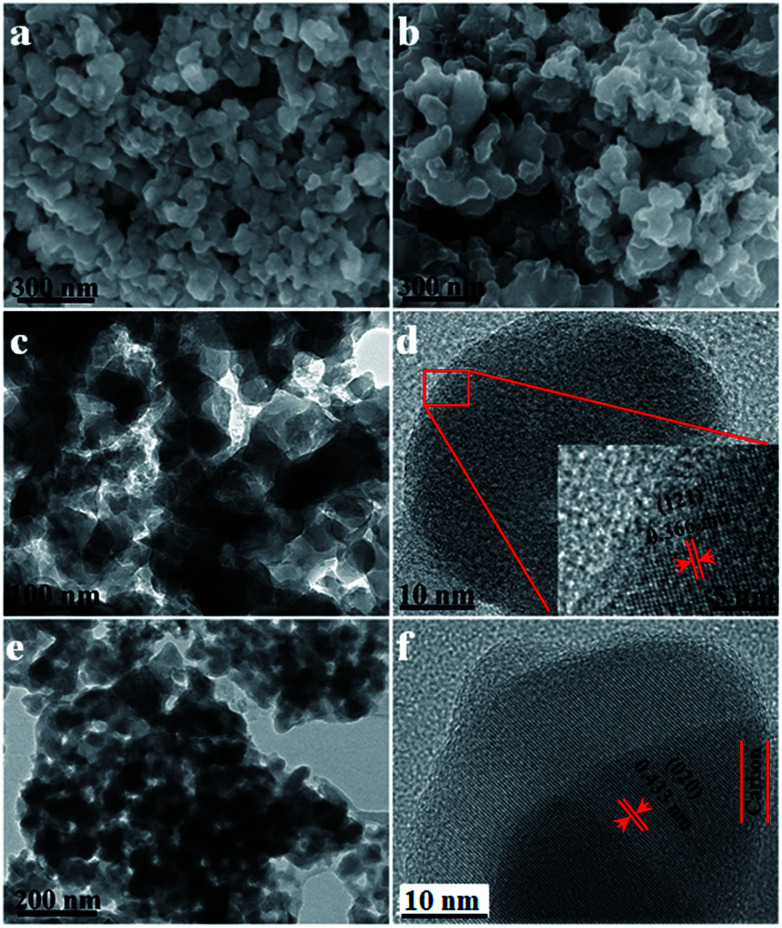
SEM images of (a) LVP and (b) LVP@C, TEM images of (c) LVP and (e) LVP@C, and HRTEM images of (d) LVP and (f) LVP@C.

As shown in the SEM images of the LVP NCs and LVP@C (Fig. 3a and b), nanoscale LVP NCs are clearly present and have a fairly uniform particle size. Meanwhile, the particles of LVP@C are connected with each other to some extent. Fig. 3c shows the TEM image of the as-synthesized LVP NCs, which display monodispersity. The grain size is about 29 nm, which is in accordance with the size calculated from the XRD pattern. Compared to other materials synthesized *via* solid-state reactions, sol–gel methods or hydrothermal syntheses,^5,34,35^ these particles display better homogeneity and smaller size, which are beneficial for ion diffusion kinetics.^15^ To further explore the structures of the LVP NCs, high-resolution TEM (HRTEM) was carried out (Fig. 3d). The clear lattice fringes demonstrate a well-crystallized structure, and the lattice spacing of 0.366 nm corresponds to the (121) plane of LVP (JDPS no. 01-072-7074). As shown in the TEM images of LVP@C (Fig. 3e and f), the LVP NCs were coated by an amorphous carbon layer converted from polydopamine. The thickness of the carbon shell was about 5 nm. The lattice fringes indicate an interplanar spacing of 0.433 nm, which corresponds to the (020) plane of LVP (JDPS no. 01-072-7074). The core–shell nanostructure of the LVP@C is beneficial for accelerating electron transfer and enhancing the lithium storage properties.^36^

**Fig. 4:**
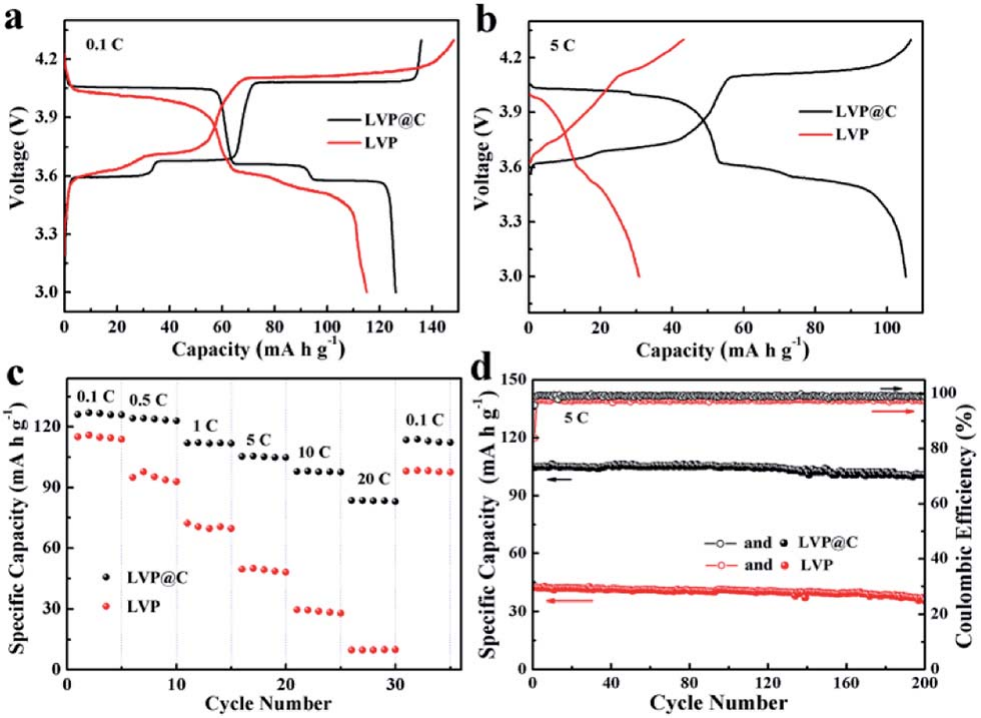
Electrochemical properties of LVP and LVP@C NCs. (a) Initial charge–discharge curves at a current rate of 0.1C in the voltage window of 3.0–4.3 V. (b) Charge–discharge curves at 5C in the voltage window of 3.0–4.3 V. (c) Rate performances from 0.1 to 20C in the voltage window of 3.0–4.3 V. (d) Cycling performance curves at a rate of 5C from 3.0 to 4.3 V.

The electrochemical properties of LVP and the LVP@C NCs were investigated between 3.0 and 4.3 V, as shown in Fig. 4. The initial discharge–charge voltage curves of LVP and the LVP@C NCs are illustrated in Fig. 4a at a low current rate of 0.1C (1C = 133 mA g^−1^). Both the discharge–charge profiles of the samples exhibit three charge plateaus and three discharge plateaus, corresponding to a sequence of phase-transition processes between the single Li_*x*_V_2_(PO_4_)_3_ (*x* = 3.0, 2.5, 2.0, and 1.0).^37^ The discharge capacity of the LVP@C NCs was 126 mA h g^−1^, which is higher than that of the LVP NCs (115 mA h g^−1^). Meanwhile, the LVP@C NCs have smaller electrode polarization than the LVP NCs. Upon increasing discharge–charge rate (*e.g.*, 5C; Fig. 4b), the electrochemical plateaus become undistinguished, and the difference in potential between the charging and discharging plateaus gradually increases due to increased electrode polarization, especially in the LVP NCs. As illustrated in Fig. 4c, the rate performances of the LVP and LVP@C NCs electrodes were tested. The LVP@C NCs showed smaller capacity decay with increasing rate compared to LVP. At a high current rate of 20C, the discharge capacity of the LVP@C NCs is 84 mA h g^−1^, which is even higher than that of the LVP NCs at 1C. Moreover, when the current density returned to 0.1C, a discharge capacity as high as 114 mA h g^−1^ (91% of the initial capacity) was retained for the LVP@C NCs, much higher than that of 98 mA h g^−1^ for the LVP NCs. In this experiment, the LVP particles are very small to nanoscale, which shortens the ion diffusion length and accelerates lithium ion diffusion; however, the poor electronic conductivity is one of the decisive properties for lithium storage. Improving the electronic conductivity by carbon coating combined with accelerated lithium ion diffusion leads to the perfect rate capability of the LVP@C NCs. To further evaluate the electrochemical properties of the obtained samples, we studied the cycling performance of the electrodes at 5C in the voltage range of 3.0–4.3 V, as shown in Fig. 4d. The initial discharge capacity of the LVP@C NCs was 105 mA h g^−1^. After 200 cycles, the capacity of the LVP@C NCs was 101 mA h g^−1^, corresponding to a capacity retention of 96.2% of its initial value. For the LVP NCs, the capacity after 200 cycles was 42.3 mA h g^−1^ (84.4% capacity retention). Fig. 4d shows that the coulombic efficiencies of both the LVP and LVP@C NCs are lowest during the first charge process and then improve after the second charge process. The decomposition of the electrolyte forms a solid electrolyte interphase (SEI) film on the electrode surface, and lithium is consumed from LVP, causing the initial charge–discharge process to be irreversible.^8^ Meanwhile, the poor electronic conductivity of pristine LVP without carbon coating prevents the full conversion of Li_3_V_2_(PO_4_)_3_ to LiV_2_(PO_4_)_3_ during the initial charge–discharge process and leads to a lower coulombic efficiency compared to the LVP@C NCs. After the second cycle, the efficiency of the LVP@C NCs remained at approximately 100%, indicating that the electrochemical Li^+^ insertion–extraction process is completely reversible, even at high rate.^13^

**Fig. 5:**
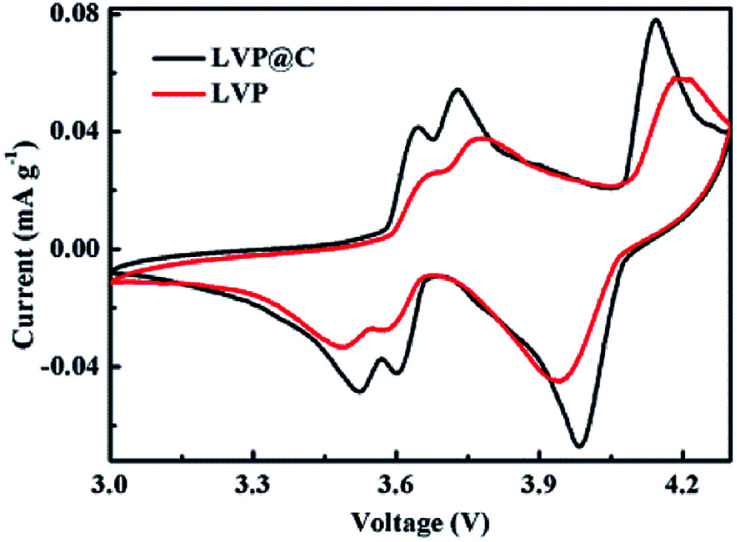
CV profiles of LVP@C NCs and LVP NCs at a scanning rate of 0.1 mV s^−1^ from 3.0 to 4.3 V.

To further investigate the electrochemical properties of the LVP and LVP@C NCs, cyclic voltammograms (CVs) were measured at a scanning rate of 0.1 mV s^−1^. As shown in Fig. 5, the CV curve of LVP@C NCs electrode is very similar with LVP NCs. The LVP@C NCs exhibits sharper and more symmetrical current peaks than LVP NCs. In addition, the anodic peaks move left, while the cathodic peaks move right compared with those of the LVP NCs, indicating less polarization of the LVP@C NCs electrodes.^38^ These results indicate that the carbon coating layer helps improve the electrochemical performance.^39,40^

The LVP@C NCs exhibit excellent electrochemical performance, which might be attributed to their novel structure. The nanosized particles reduce the diffusion distance for the lithium ions. Meanwhile, the coated carbon enhances electron transport in the charge–discharge process. That is to say, the conductive carbon layer and smaller particle size of the electrode are favorable for the lithium storage properties. Meanwhile, the worse charge and discharge capabilities of the uncoated LVP NCs demonstrate that both the diffusion of lithium ions and electron conductivity are the decisive factors in the electrochemical performance of the electrode.

## Conclusions

In summary, we report a facile two-step method to synthesize LVP@C NCs with improved lithium storage properties. The preparation includes a liquid-phase synthesis of LVP NCs using high-boiling-point organic solution and a subsequent carbon-coating process. The formation mechanism of the NCs may be based on OA and OAm acting as capping agents to limit the sizes of crystal nuclei to the nanoscale. Crystallization cannot be achieved in the organic-phase environment (at 320 °C); thus, calcination is essential to prepare LVP. The nanosized particles accelerate lithium ion diffusion, and the carbon coating enhances the electronic conductivity of LVP. Therefore, both the intrinsic problems of the LVP electrode are solved. The resultant LVP@C NCs exhibit perfect rate performance (84 mA h g^−1^ at a rate of 20C) and outstanding cycling stability (96.2% capacity retention after 200 cycles at 5C). These results prompt us to further investigate the synergistic effects between lithium ion diffusivity and electronic conductivity as a next step in developing high-performance lithium-ion batteries. We anticipate that this effective strategy can be further applied to other anode and cathode materials to boost electrochemical performance.

## Conflicts of interest

There are no conflicts to declare.

## Supplementary Material
